# SPTBN2 suppresses ferroptosis in NSCLC cells by facilitating SLC7A11 membrane trafficking and localization

**DOI:** 10.1016/j.redox.2024.103039

**Published:** 2024-01-14

**Authors:** Jun Deng, Xu Lin, Jiajia Qin, Qi Li, Yingqiong Zhang, Qingyi Zhang, Cong Ji, Shuying Shen, Yangling Li, Bo Zhang, Nengming Lin

**Affiliations:** a Key Laboratory of Clinical Cancer Pharmacology and Toxicology Research of Zhejiang Province, Affiliated Hangzhou First People's Hospital, School of Medicine, Zhejiang University, Hangzhou, 310006, China; b Department of Pharmacy, The First Affiliated Hospital of Guangxi Medical University, GuangXi, 530021, China; c Department of Thoracic Surgery, The First Affiliated Hospital, Zhejiang University School of Medicine, Hangzhou, 310003, China; d Department of Pharmacy, The second Affiliated Hospital of Guangxi Medical University, GuangXi, 530007, China; e School of Pharmaceutical Sciences, Zhejiang Chinese Medical University, Hangzhou, 311402, China; f Westlake Laboratory of Life Sciences and Biomedicine of Zhejiang Province, Westlake University, Hangzhou, 310024, China; g Cancer Center, Zhejiang University, Hangzhou, 310058, China

**Keywords:** SPTBN2, Ferroptosis, NSCLC, SLC7A11, membrane trafficking

## Abstract

The function of SLC7A11 in the process of ferroptosis is well-established, as it regulates the synthesis of glutathione (GSH), thereby influencing tumor development along with drug resistance in non-small cell lung cancer (NSCLC). However, the determinants governing SLC7A11's membrane trafficking and localization remain unknown. Our study identified SPTBN2 as a ferroptosis suppressor, enhancing NSCLC cells resistance to ferroptosis inducers. Mechanistically, SPTBN2, through its CH domain, interacted with SLC7A11 and connected it with the motor protein Arp1, thus facilitating the membrane localization of SLC7A11 — a prerequisite for its role as System Xc^−^, which mediates cystine uptake and GSH synthesis. Consequently, SPTBN2 suppressed ferroptosis through preserving the functional activity of System Xc^−^ on the membrane. Moreover, Inhibiting SPTBN2 increased the sensitivity of NSCLC cells to cisplatin through ferroptosis induction, both in vitro and in vivo. Using Abrine as a potential SPTBN2 inhibitor, its efficacy in promoting ferroptosis and sensitizing NSCLC cells to cisplatin was validated. Collectively, SPTBN2 is a potential therapeutic target for addressing ferroptosis dysfunction and cisplatin resistance in NSCLC.

## Highlights

• SPTBN2 is a suppressor of ferroptosis acting on System Xc^−^.
• SPTBN2 binds to SLC7A11 through its CH domain and facilitates the membrane trafficking and localization of SLC7A11.
• SPTBN2 is a potential target against cisplatin resistance.
• Abrine is an effective inhibitor of SPTBN2.

## Graphical abstract

**Figure:**
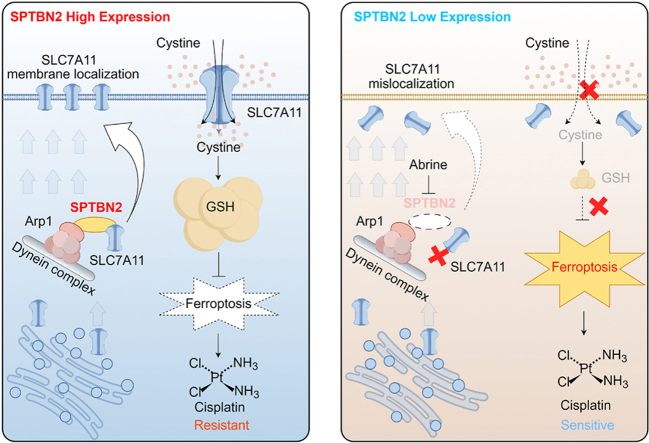

## Abbreviations

GSH: Glutathione
NSCLC: Non-small cell lung cancer
SPTBN2: Spectrin Beta, Non-Erythrocytic 2
CH domain: Calponin homology domain
SR: Spectrin repeat
PH domain: Pleckstrin homology domain
KO: knockout
Fer-1: Ferrostatin-1
Z-VAD: Z-VAD-FMK
Nec-1s: Necrostatin-1
OE: overexpression
NAC: Acetylcysteine
Co-IP/MS: Co-Immunoprecipitation/Mass Spectrometry
BFA: Brefeldin A
PFS: Progression-Free Survival
HE staining: hematoxylin and eosin staining

## 1 Introduction

Ferroptosis is an iron-dependent programmed cell death characterized by lipid peroxide accumulation [[1], [2], [3]]. This process is modulated by System Xc^−^, a cystine/glutamate antiporter in the cell membrane composed of two subunits: SLC7A11 (transports cystine and glutamate) and SLC3A2 (stabilizes SLC7A11) [4]. SLC7A11, the core subunit of System Xc^−^, maintains cellular redox balance by importing cystine for glutathione (GSH) synthesis, a key antioxidant preventing lipid peroxidation, thus reducing lipid peroxide levels and protecting against ferroptosis [5].The function and regulation of SLC7A11, which is primarily localized to the plasma membrane, are affected by its membrane localization [[6], [7], [8]]. However, the determinants governing its trafficking and localization to the cell membrane surface remain unknown.

Spectrin Beta, Non-Erythrocytic 2 (SPTBN2) is a vital constituent of the membrane-cytoskeleton interface, characterized by a structure encompassing two N-terminal calponin homology (CH) domains, 17 spectrin repeat (SR) sequences, and a C-terminal pleckstrin homology (PH) domain [9]. As a membrane cytoskeleton protein, SPTBN2 plays a crucial role in preserving the functionality of membrane proteins. For instance, excitatory amino acid transporter 4 (EAAT4), a member of the glutamate transporter family which is primarily characterized by its extrasynaptic localization on perisynaptic membranes near release sites, requires a direct interaction with SPTBN2 to maintain its membrane localization capability [10]. Stankewich et al. showed that a loss of SPTBN2 function led to a significant decrease of EAAT4 on the membrane surface, causing EAAT4 to accumulate in the Golgi region [11]. While considerable evidence links various cell surface membrane proteins, such as SLC7A11, transferrin receptor, and ferritin receptor, with the regulation of ferroptosis [[12], [13], [14]], the role of SPTBN2, a membrane protein function regulator, in ferroptosis remains unexplored and merits further investigation.

The significance of ferroptosis in the progression and drug resistance of non-small cell lung cancer (NSCLC) has garnered research traction in recent years. Liu et al. demonstrated that the inhibition of NRF2 could enhanced the therapeutic effect on NSCLC both in vitro and in vivo by inducing ferroptosis [15]. In a separate study, Wang et al. revealed that the combination of nanocatalytic sensitizers and AHP-DRI-12 could overcome AZD9291 resistance and metastasis of NSCLC through the induction of ferroptosis and multi-target interference [16].Cisplatin, a first-line chemotherapeutic agent for NSCLC, induces ferroptosis by depleting GSH [[17], [18], [19]]. Abnormal GSH synthesis in tumor cells can lead to both ferroptosis dysfunction and cisplatin resistance [20]. Therefore, targeting ferroptosis is a potentially effective strategy for overcoming cisplatin resistance in NSCLC cells. However, precise mechanisms by which ferroptosis is regulated at the cellular level remain unclear and merit in-depth studies.

In this study, we found that SPTBN2 suppressed ferroptosis by maintaining the function of System Xc^−^ in NSCLC cells. Specifically, SPTBN2 bound to SLC7A11 through its CH domain, and connected SLC7A11 with Arp1, a key molecule of the dynactin complex, which facilitated the transport of SLC7A11 from the cytoplasm to the membrane. Furthermore, SPTBN2 inhibition sensitized NSCLC cells to cisplatin by inducing ferroptosis in vitro and in vivo. The validation of SPTBN2 as a suppressor of ferroptosis opens new avenues for the development of ferroptosis-based cancer therapies.

## 2 Results

### 2.1 SPTBN2 suppresses ferroptosis through System Xc^-^

Through preliminary experiments, we established SPTBN2 as a potential oncogene, which was upregulated and correlated with poor prognosis in patients with NSCLC (Supplementary Figs. 1A–I). To gain deeper insights into the role of SPTBN2 in NSCLC, we performed KEGG/GO enrichment analysis of SPTBN2 using the TCGA datasets. The analysis revealed SPTBN2 was significantly enriched in the ferroptosis pathway (Supplementary Figs. 2A and B), suggesting a potential involvement of SPTBN2 in ferroptosis. To validate this hypothesis, we created stable SPTBN2 knockout (KO) cell lines in H358 and A549 using CRISPR-Cas9 technology and exposed them to various programmed cell death inducers (including apoptosis, autophagy, necrosis, pyroptosis, and ferroptosis). Our findings indicated that SPTBN2 KO enhanced the sensitivity of NSCLC cells to ferroptosis inducers such as FIN56, RSL3, and Erastin, without affecting the efficacy of other cell death inducers (Fig. 1A). Notably, RSL3 and Erastin are specific inhibitors for Glutathione peroxidase 4 (GPX4) and System Xc^−^, respectively, while FIN56 is known to exert inhibitory effects on both GPX4 and coenzyme Q10 (CoQ10) [21]. The ferroptosis inhibitor Ferrostatin-1 (Fer-1), but not other cell death inhibitors (Z-VAD or Nec-1s), reversed the sensitization effect of FIN56 on SPTBN2 KO cells (Fig. 1B, Supplementary Fig. 2C). Classic biomarkers of ferroptosis, MDA and lipid ROS, accumulated in SPTBN2 KO cells following low-dose FIN56 treatment (Fig. 1C, Supplementary Fig. 2E; Fig. 1D, Supplementary Fig. 2D). Morphological changes in cells and organelles, another indicator of ferroptosis, were observed by microscopic examinations. Consistent with the trend for lipid ROS, optical microscopy revealed significant organelle shrinkage in SPTBN2 KO cells after low-dose FIN56 treatment (Fig. 1E). Transmission electron microscopy confirmed that SPTBN2 KO enhanced mitochondrial shrinkage and increase in membrane density induced by FIN56 (Fig. 1F). Collectively, our data suggest that SPTBN2 KO potentiates the induction of ferroptosis following stimulation.

**Fig. 1:**
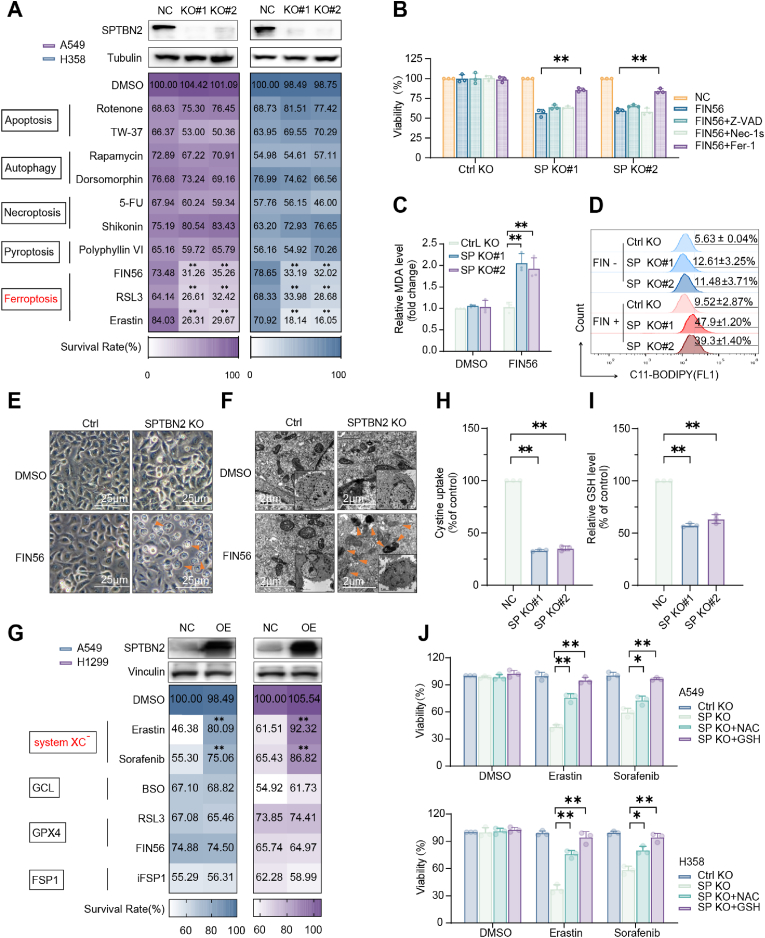
SPTBN2 suppresses ferroptosis via the System Xc^−^ Pathway A: Cell viability assay of SPTBN2 KO NSCLC cells following 48 h co-culture with various programmed cell death inducers. Data are the average of three biological replicates, each in technical triplicate. Statistical analysis was performed using a two-tailed, unpaired Student's t-test; **p < 0.01, vs. NC. Specific values: A549 (FIN56 + NC, 73.48 ± 2.74 %; FIN56 + KO#1, 31.26 ± 1.91 %; FIN56 + KO#2, 35.26 ± 2.04 %; RSL3+NC, 64.14 ± 1.42 %; RSL3+KO#1, 26.61 ± 2.43 %; RSL3+KO#2, 32.42 ± 4.39 %; Erastin + NC, 84.03 ± 3.25 %; Erastin + KO#1, 26.31 ± 2.45 %; Erastin + KO#2, 29.67 ± 4.80 %), H358 (FIN56 + NC, 78.65 ± 0.88 %; FIN56 + KO#1, 33.19 ± 2.78 %; FIN56 + KO#2, 32.02 ± 3.34 %; RSL3+NC, 68.33 ± 3.77 %; RSL3+KO#1, 33.98 ± 3.87 %; RSL3+KO#2, 28.68 ± 3.30 %, Erastin + NC, 70.92 ± 3.49 %; Erastin + KO#1, 18.14 ± 3.04 %; Erastin + KO#2, 16.05 ± 3.27 %). B: Cell viability assay of SPTBN2 KO A549 cells co-cultured with indicated programmed cell death inhibitors under a low dose of FIN56 (0.6 μM) for 48 h. Z-VAD: apoptosis inhibitor Z-VAD-fmk (20 μM); Nec-1s: necroptosis inhibitor necrostatin-1s (2 μM); Fer-1: ferroptosis inhibitor ferrostatin-1 (1 μM); SP: SPTBN2. C–F: Analysis of MDA content (C), lipid peroxidation level via flow cytometry (D), cell morphology via optical microscopy (E), and subcellular organelle structure via transmission electron microscopy (F) in SPTBN2 KO A549 cells induced by FIN56 (0.6 μM) for 48 h. Orange arrows in (E) and (F) indicate ferroptotic cell morphology and mitochondrial shrinkage during ferroptosis, respectively. G: Cell viability assay of SPTBN2 overexpressing A549 and H1299 cells co-cultured with different pathway ferroptosis inducers for 48 h. The data represent mean values obtained from three independent experiments, each performed in triplicate. Statistical significance was determined using a two-tailed, unpaired Student's t-test. The symbols **p < 0.01 denote statistical significance compared to the NC group. Specific mean values with standard deviations are as follows: In A549 cells, viability was 46.38 ± 2.48 % with Erastin + NC and increased to 80.09 ± 6.86 % with Erastin + OE; Sorafenib + NC showed 55.30 ± 1.78 % viability, and Sorafenib + OE exhibited 75.06 ± 1.33 %. In H358 cells, viability was 61.51 ± 1.45 % with Erastin + NC and increased to 92.32 ± 3.64 % with Erastin + OE; Sorafenib + NC showed 65.43 ± 2.72 % viability, and Sorafenib + OE was 86.82 ± 2.93 %. H–I: Cystine uptake level (H) and GSH level (I) of SPTBN2 KO A549 cells. J: Cell survival assay of SPTBN2 KO NSCLC cells co-cultured with low dose of System Xc^−^ ferroptosis inducers (Erastin 1 μM; Sorafenib 5 μM) with or without NAC (4 mM), GSH (4 mM) treatment for 48 h (*p < 0.05, **p < 0.01). (For interpretation of the references to colour in this figure legend, the reader is referred to the Web version of this article.)

We subsequently investigated how SPTBN2 regulates ferroptosis. We used lentiviral plasmid to establish SPTBN2 overexpression (OE) stable transfection cell lines in H1299 and A549. Cells were co-cultured with ferroptosis inducers targeting diverse pathways (including System Xc^−^, GCL, GPX4, and FSP1). The overexpression of SPTBN2 partially reversed the ferroptosis induced by Erastin and Sorafenib, but not by inducers targeting GCL, GPX4, and FSP1 (Fig. 1G). This suggested that SPTBN2 specifically modulated ferroptosis through System Xc^−^. Moreover, cystine uptake (Fig. 1H, Supplementary Fig. 2G) and GSH synthesis (Fig. 1I, Supplementary Fig. 2F), critical functions of System Xc^−^, were diminished significantly following SPTBN2 KO. The downstream products of System Xc^−^, Acetylcysteine (NAC) and GSH, markedly alleviated ferroptosis induction in SPTBN2 KO cells following ferroptosis stimulation (Fig. 1J). Taken together, SPTBN2 is a negative regulator of ferroptosis and targets System Xc^−^.

### 2.2 SPTBN2 interacts with SLC7A11 through its CH domain

To explore the molecular mechanism underlying the regulation of System Xc^−^ by SPTBN2, we performed co-immunoprecipitation/mass spectrometry (Co-IP/MS) to identify proteins that interacted with SPTBN2. Intriguingly, an interaction between SPTBN2 and SLC7A11, the functional subunit of System Xc^−^, was found (Fig. 2A). This interaction was further confirmed by endogenous (Fig. 2B, Supplementary Fig. 3A) and exogenous (Fig. 2C) Co-IP/western blotting. Additionally, we examined the cellular localization of SPTBN2 and SLC7A11 using immunofluorescence and laser confocal microscopy, revealing colocalization (R = 0.78) of SPTBN2 and SLC7A11 on the cell membrane and partially in the cytoplasm (Fig. 2D). To identify the region of SPTBN2 responsible for this interaction, we co-expressed various Flag-tagged SPTBN2 fragments (Fig. 2E) with SLC7A11 and performed Co-IP/western blotting. We found that SPTBN2 fragment 1-304aa, which contained the CH domain, bound to SLC7A11 (Fig. 2F). Subsequently, we constructed a three-dimensional structural model of the complex using AutoDockTools (1.5.7) based on X-ray crystallography. Docking simulation data revealed that the amino acids CYS272, ASN280, MET283, GLN263, SER265, THR249, SER265, LYS191 of SPTBN2 interacted with SLC7A11 (Fig. 2G). Consistently, these amino acids were all located in the CH domain within the 1-304aa fragment. These data suggest that the CH domain of SPTBN2 mediates its interaction with SLC7A11.

**Fig. 2:**
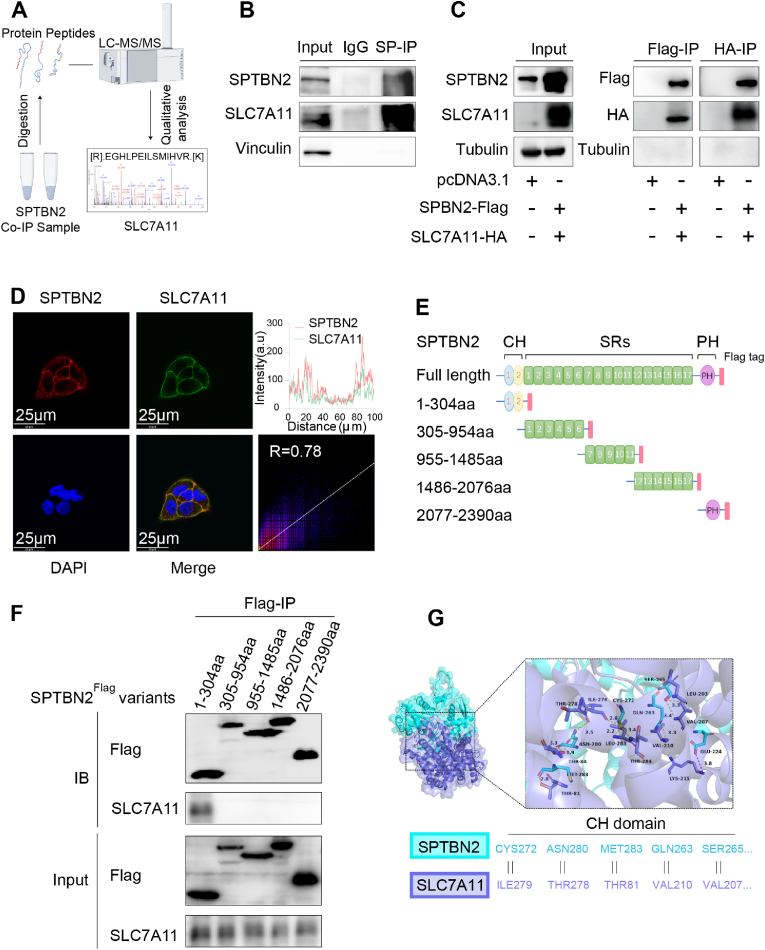
SPTBN2 interacts with SLC7A11 through CH domain A: Co-immunoprecipitation (Co-IP) of endogenous SPTBN2 in A549 cells using an anti-SPTBN2 antibody, followed by mass spectrometry analysis revealing the presence of peptide fragments of SLC7A11 in the SPTBN2 Co-IP complex. B: Endogenous Co-IP/western blotting experiment performed in A549 cells using an SPTBN2 antibody. C: Transfection of Flag-tagged SPTBN2 and HA-tagged SLC7A11 into 293T cells, followed by exogenous Co-IP experiments with Flag and HA antibodies, respectively. D: Immunofluorescence and laser confocal microscopy used to observe the co-localization of SPTBN2 and SLC7A11 in A549 cells. Co-localization wave-pop correlation analysis was conducted using Image J. E: Schematic representation of full-length and truncated mutants of SPTBN2. F: Transfection of truncated mutants of SPTBN2 in 293T cells, followed by exogenous Co-IP/western blotting experiments using Flag antibody-coated magnetic beads. G: Computer simulation model of molecular docking between SPTBN2 and SLC7A11, created using AutoDockTools (1.5.7).

### 2.3 SPTBN2 maintains the activity of System Xc^−^ by promoting the localization of SLC7A11 on the cell membrane

Transcriptional inhibition and protein degradation are two major causes for SLC7A11 functional loss [22]. We investigated the effect of SPTBN2 KO on the mRNA expression and protein stability of SLC7A11 through RT-qPCR and western blotting, respectively. Our findings indicated that SPTBN2 KO did not affect the mRNA levels (Supplementary Fig. 3B) or protein stability (Supplementary Fig. 3C) of SLC7A11 significantly. As a membrane protein, proper membrane localization is essential for the functionality of SLC7A11 [23]. We then assessed whether SPTBN2 KO altered the membrane localization of SLC7A11. Immunofluorescence analysis demonstrated that, in control cells, SLC7A11 was primarily localized at the cell membrane (Fig. 3A). Conversely, in SPTBN2 knockout (KO) cells, SLC7A11 showed diffused cytoplasmic distribution. Intriguingly, SPTBN2 deficiency prevented the membrane localization of SLC7A11, even after overexpressing SLC7A11. Only through co-expression with SPTBN2 did SLC7A11 regain membrane localization, as confirmed by the membrane protein extraction assay (Fig. 3B), flow cytometry for SLC7A11 proteins on the cell surface (Fig. 3C), and in vivo animal experiments (Fig. 3D). These results consistently confirmed the regulatory role of SPTBN2 in the membrane localization of SLC7A11. Subsequent investigations revealed that SPTBN2 affected the activity of System Xc^−^ by modulating the membrane localization of SLC7A11. Notably, SPTBN2 KO diminished cystine uptake (Fig. 3E) and GSH synthesis (Fig. 3F). Only overexpressing SLC7A11 failed to restore cystine uptake and GSH levels in the absence of SPTBN2, with significant recovery observed only following SPTBN2 co-expression. Flow cytometry analysis using the fluorescent probe, C11-BODIPY (Fig. 3G), and cell viability assays (Fig. 3H) consistently revealed the pattern of lipid peroxidation and ferroptosis dictated by SLC7A11 membrane localization. Taken together, SPTBN2 KO impairs the function of SLC7A11 by disrupting its membrane localization, thereby promoting ferroptosis.

**Fig. 3:**
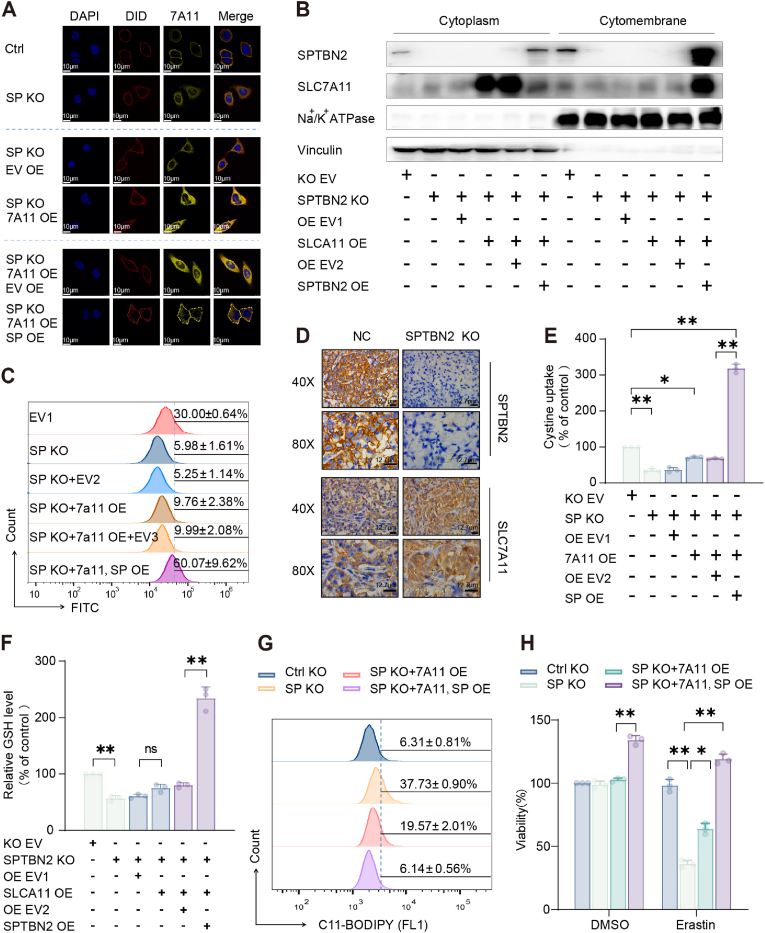
SPTBN2 maintains System Xc^−^ activity by promoting the cell membrane surface localization of SLC7A11 A–C: Following specified gene intervention (KO or OE) of SPTBN2 and SLC7A11, respectively, immunofluorescence and laser confocal microscopy were used to observe SLC7A11 localization in A549 cells. DAPI: nuclear dye; DID: membrane dye; 7A11: SLC7A11, SP: SPTBN2; Scale bars (inset), 10 μm (A). Membrane protein and cytoplasmic protein were separated, followed by a western blotting experiment (B). Flow cytometry was used to label the SLC7A11 cell surface antigen and detect the expression level of SLC7A11 on the cell membrane surface (C). D: Immunohistochemistry detection of SPTBN2 and SLC7A11 protein expression and localization in subcutaneous xenograft model of A549 and A549 KO cells; E–F: Following specified gene intervention (KO or OE) of SPTBN2 and SLC7A11, respectively, Cystine uptake level (E), GSH content (F) were conducted in A549 cells (*p < 0.05, **p < 0.01). G–H: Following specified gene intervention (KO or OE) of SPTBN2 and SLC7A11, respectively, A549 cells were co-cultured with or without Erastin (1 μM) for 48 h. Subsequently, lipid peroxidation level via flow cytometry (G), and cell survival assay (H) were conducted (**p < 0.01).

### 2.4 SPTBN2 facilitates the trafficking and localization of SLC7A11 through the interaction of SLC7A11 and Arp1

To elucidate the direct mechanism by which SPTBN2 regulated the membrane localization of SLC7A11, we generated a CH domain deletion mutant of SPTBN2, namely Del aa2-303 (Fig. 4A). Mutant SPTBN2 failed to bind to SLC7A11 after overexpression in cells (Fig. 4B). Given that the Golgi apparatus is the gateway for the transport of cell surface proteins, and certain membrane proteins require processing by the Golgi apparatus before reaching the cell surface [24], we investigated whether SPTBN2 affected Golgi-mediated transport of SLC7A11. Immunofluorescence analysis revealed that SPTBN2 OE restored the membrane localization of SLC7A11 in A549^SPTBN2−/SLC7A11+^ cells, while overexpression of mutant SPTBN2 (Del aa2-303) did not. Notably, treatment with the inhibitor of the Golgi apparatus, Brefeldin A (BFA), eliminated the effect of SPTBN2 OE, thus preventing SLC7A11 from reaching the membrane (Fig. 4C). Western blotting results were consistent with those of immunofluorescence analysis (Fig. 4D, Supplementary Fig. 4A), indicating that SPTBN2 regulated the membrane localization of SLC7A11 before, rather than after, the Golgi transport. Given previous reports of SPTBN2 binding to the dynactin complex, acting as a transport enhancer, and facilitating the subcellular localization of other proteins [25,26], the role of SPTBN2 as an auxiliary protein in the membrane transport of SLC7A11 was hypothesized. To test this hypothesis, Co-IP/MS results for SPTBN2 and SLC7A11 were subjected to intersection analysis, leading to the identification of 57 proteins (Supplementary Table 1). Among these proteins, Arp1 was remarkable (Fig. 4E). Arp1 is a pivotal component of the dynactin family, serving as an intracellular motor responsible for the transport and localization of organelles and proteins [27,28]. We first verified the interaction of SPTBN2, SLC7A11, and Arp1 by Co-IP/western blotting (Fig. 4F). Subsequently, gene knockout experiments revealed that SPTBN2 deficiency disrupted the interaction between Arp1 and SLC7A11, while the deficiency of SLC7A11 or Arp1 did not affect the interaction of SPTBN2 with the other protein (Fig. 4G, Supplementary Fig. 4B). Collectively, SPTBN2 can independently bind to SLC7A11 and Arp1, thereby acting as a “bridge” to link Arp1 and SLC7A11. This interaction forms a multi-component complex, assisting SLC7A11 in its transport to the membrane with the help of dynactin. The schematic of this mechanism is shown in Fig. 4H.

**Fig. 4:**
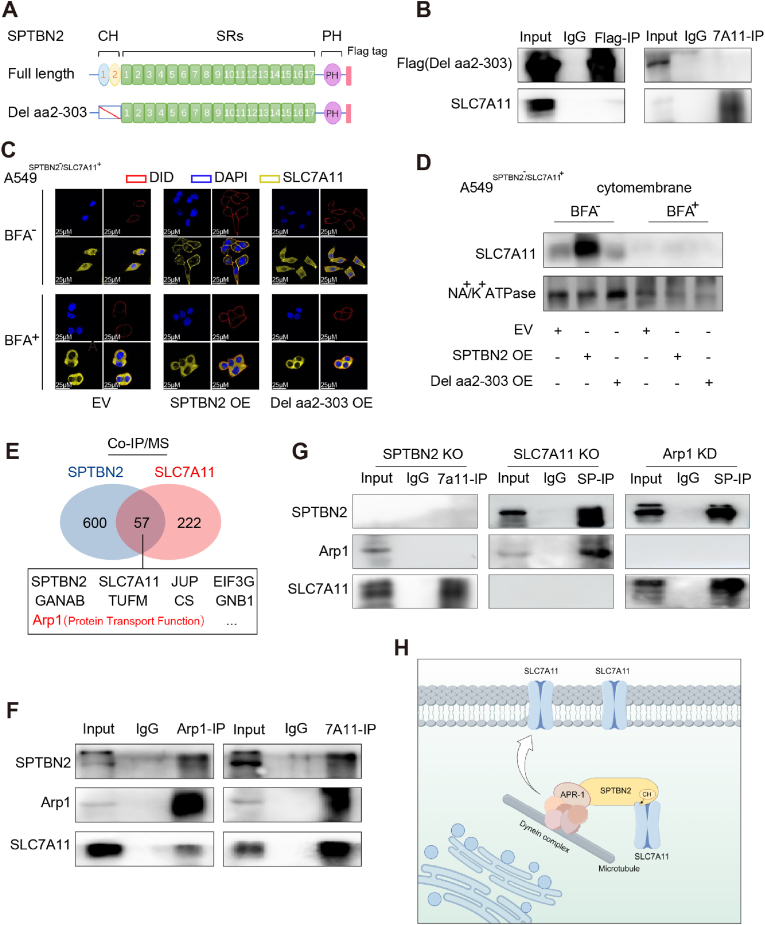
SPTBN2 facilitates the cell membrane trafficking and localization of SLC7A11 by connecting SLC7A11 and Arp1. A: Schematic representation of CH domain deletion mutant (Del aa2-303) of SPTBN2. B: Transfection of Flag-tagged CH domain deletion mutant (Del aa2-303) of SPTBN2 in A549, followed by a Co-IP experiment with Flag and SLC7A11 antibodies, respectively. 7A11: SLC7A11. C–D: Overexpression of full-length SPTBN2 or CH domain deletion mutant (Del aa2-303) of SPTBN2 in A549^SPTBN2−/SLC7A11+^ cells, followed by co-culture with or without BFA (100 nM) for 24 h. Immunofluorescence and laser confocal microscopy were used to observe SLC7A11 localization in cells. DAPI: nuclear dye; DID: membrane dye (C); Membrane protein was extracted, and western blotting was used to detect the expression level of SLC7A11 protein in the cell membrane (D). E. Intersection of Co-IP/MS results for SPTBN2 and SLC7A11 identified a total of 57 proteins, including Arp1. F. Endogenous Co-IP/western blotting experiment conducted in A549 cells using Arp1 and SLC7A11 antibodies, respectively. 7A11: SLC7A11. G. Sequential knockout of SPTBN2, SLC7A11, and Arp1 in A549 cells, followed by a Co-IP/western blotting experiment with SLC7A11 and SPTBN2 antibodies, respectively. 7A11: SLC7A11; SP: SPTBN2. H. Schematic representation of the mechanism by which SPTBN2 assists SLC7A11 in cell membrane transport and localization.

### 2.5 Inhibition of SPTBN2 sensitizes NSCLC cells to cisplatin by promoting ferroptosis

Cisplatin is a chemotherapeutic agent closely related to ferroptosis in the current first-line treatment for NSCLC, since it binds to GSH which is an antioxidant that inhibits ferroptosis [29,30]. Given the role of SPTBN2 in GSH synthesis regulation, we investigated its effect on cisplatin resistance. Analysis using the GDSC database revealed a correlation between high SPTBN2 expression and increased cisplatin resistance (Fig. 5A). Moreover, progression-free survival (PFS) is a crucial indicator of treatment outcomes, with a diminished PFS often being associated with the ineffectiveness of adjuvant chemotherapy following surgery [31]. KM plot of patients with NSCLC confirmed that elevated SPTBN2 expression was correlated with worse PFS prognosis in both the total PFS (Fig. 5B) and chemotherapy subgroup (Fig. 5C). In order to further confirm these findings, cisplatin-resistant cell lines (A549 R2-A549 R6, H358R, and H460R) were generated (Supplementary Fig. 5I) and resistance was confirmed (Fig. 5D, Supplementary Figs. 5A–B). Besides, Resistance in A549 R6 was further confirmed in a xenograft mouse model. (Supplementary Fig. 5C). GSH content (Fig. 5E) and the cellular tolerance to ferroptosis (Fig. 5F) escalated concurrently with their growing resistance to cisplatin in NSCLC cells (Supplementary Figs. 5E–G), indicating that resistance to ferroptosis occurs in tandem with the development of cisplatin resistance. Additionally, The protein expression of SPTBN2 was correlated with resistance (Fig. 5G, Supplementary Fig. 5D). Knocking down SPTBN2 effectively reversed cisplatin resistance (Fig. 5H, Supplementary Fig. 5H), and this effect was attenuated after treatment with a ferroptosis inhibitor, Fer-1, or GSH (Fig. 5I). These findings suggest that inhibition of SPTBN2 reverses cisplatin resistance in NSCLC cells by promoting ferroptosis.

**Fig. 5:**
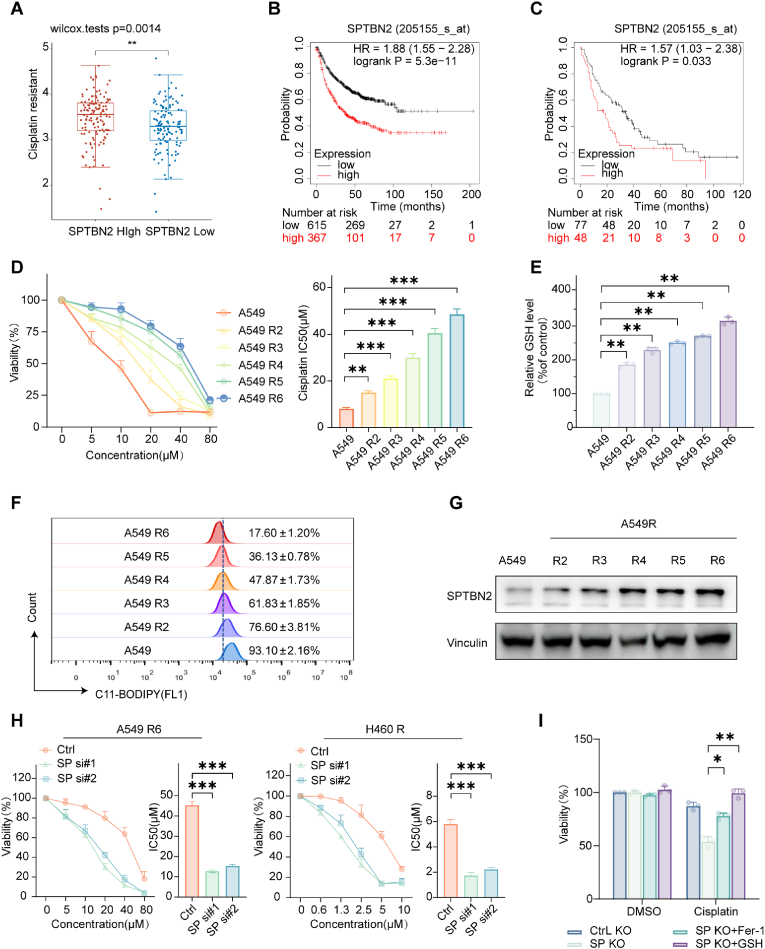
Inhibition of SPTBN2 sensitizes NSCLC cells to cisplatin by promoting ferroptosis. A: Collection of gene expression information from TCGA database and drug sensitivity information from GDSC database, followed by an analysis of the relationship between SPTBN2 expression and cisplatin drug sensitivity using the R package pRRophetic. B–C: PFS survival curves of NSCLC patients (B) and a subgroup of NSCLC patients undergoing chemotherapy (C) based on SPTBN2 expression level, using the Kaplan-Meier plotter online database. (http://kmplot.com/analysis/index.php?p=service&cancer=lung). Grouping by “auto select best cutoff”. D: Cell viability and IC50 calculation of A549 and A549 cisplatin-resistant cells (R2-R6) following treatment with cisplatin (0–80 μM) for 48 h. E: GSH level of A549 and A549 cisplatin-resistant cells (R2-R6). F: Lipid peroxidation level of A549 and A549 cisplatin-resistant cells (R2-R6) measured by flow cytometry after treatment with cisplatin (10 μM) for 48 h. G: Protein expression of SPTBN2 in A549 and A549 cisplatin-resistant cells (R2-R6). H: Cell viability and IC50 calculation of A549R and H460R after knockdown of SPTBN2 with siRNA and co-culture with cisplatin (0–80 μM) for 48; SP: SPTBN2. I: Cell survival assay of SPTBN2 KO A549R treated with cisplatin (10 μM) and co-cultured with or without Fer-1, GSH for 48 h (*p < 0.05, **p < 0.01, ***p < 0.001).

### 2.6 Abrine sensitizes NSCLC cells to cisplatin by inhibiting SPTBN2 and promoting ferroptosis

Our findings establish SPTBN2 as a potential therapeutic target for NSCLC, enhancing the efficacy of cisplatin. However, specific inhibitors of SPTBN2 are currently unavailable. Through network pharmacology methods based on the principles of network-based inference (NBI), Abrine was identified as a potential SPTBN2 inhibitor (Fig. 6A). We obtained the CAS No.: 526-31-8 of Abrine from the PubChem database (Fig. 6B) and its crystal structure from the PDB database, and performed docking with SPTBN2. The results indicated that Abrine could bind to the SPTBN2 protein target with visible hydrogen bonds and strong electrostatic interactions, with a binding energy of −5.9 kcal/mol (Fig. 6C). The inhibitory effect of Abrine on the protein level of SPTBN2 was confirmed by western blotting using different concentrations. Abrine could significantly inhibit the protein expression of SPTBN2 at doses higher than 60 μM (Fig. 6D). Additionally, Abrine did not inhibit the total protein expression of SLC7A11 (Supplementary Fig. 6C). However, it significantly suppressed the expression of SLC7A11 protein on the cell membrane at a concentration of 60 μM, an effect rescuable by SPTBN2 overexpression (Fig. 6E). In concordance, Abrine significantly reduced GSH content (Fig. 6F) and increased MDA (Fig. 6G) and lipid ROS (Fig. 6H) levels in A549 and A549R cells, which could be reversed by overexpressing SPTBN2. Therefore, we conclude that Abrine promotes ferroptosis by inhibiting the expression of SPTBN2. To evaluate the synergistic effect of Abrine and cisplatin, we co-treated A549 and A549R cells with different concentrations of abrine and cisplatin (Fig. 6I). Although Abrine exhibited a sensitizing effect toward cisplatin in both cell types, it was more pronounced in A549R (CI value = 0.82 ± 0.09) than in A549 cells (0.47 ± 0.16). This effect could be alleviated by overexpressing SPTBN2 or through treatment with the ferroptosis inhibitor, Fer-1, or GSH (Fig. 6J). Taken together, Abrine sensitizes NSCLC cells to cisplatin by downregulating SPTBN2 and promoting ferroptosis.

**Fig. 6:**
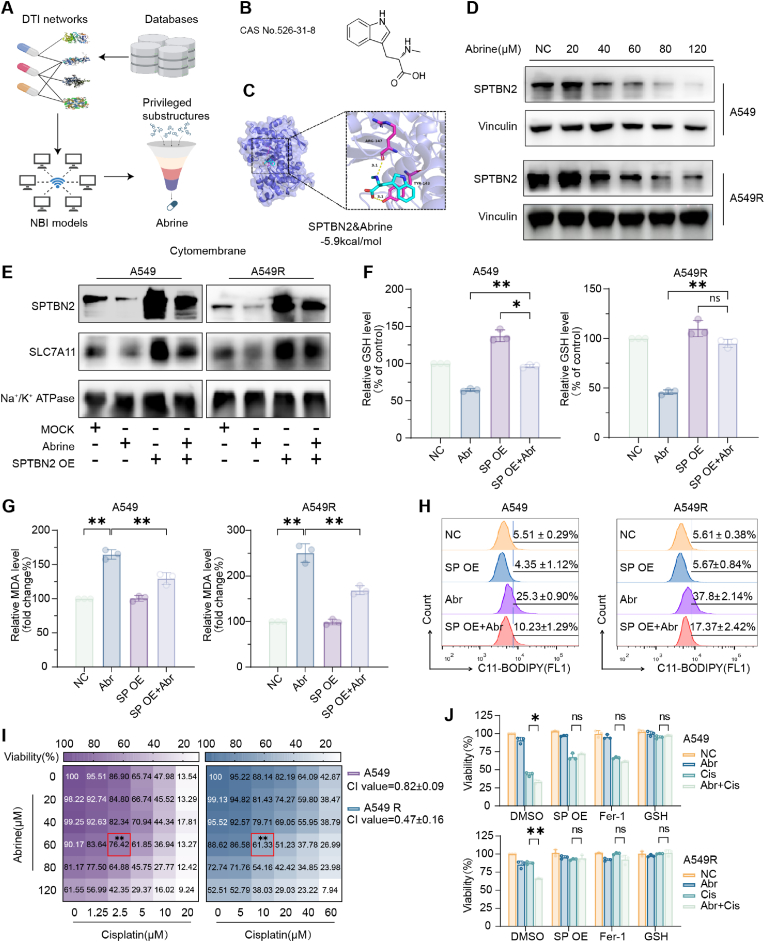
Abrine sensitizes NSCLC cells to cisplatin by inhibiting SPTBN2 and promoting ferroptosis. A: Identification of potential inhibitors of SPTBN2 using network pharmacology techniques based on the NBI methods. B: Chemical structure of abrine. C: Three-dimensional structure of the molecular docking of Abrine and SPTBN2, with a binding energy of −5.9 kcal/mol. D: Protein expression level of SPTBN2 as determined by western blotting following treatment with Abrine (20–120 μM) for 72 h in indicated cells. E–H: Overexpression of SPTBN2 in indicated cells, followed by treatment with Abrine (60 μM) for 72 h. Membrane protein was extracted, and western blotting was used to detect the protein expression level of SPTBN2 and SLC7A11 on the cell membrane (E). GSH content (F), MDA level (G), and lipid peroxidation level (H) were also determined. Data in Fig. 6H represent mean ± SD of three biological replicates. SP: SPTBN2; Abr: Abrine. I: Cell viability as determined by the CCK8 method following co-culture with Abrine (0–120 μM) and cisplatin (0–60 μM) for 48 h in indicated cells. Data are the average of three biological replicates, each in technical triplicate. Statistical comparisons were performed using a two-tailed, unpaired Student's t-test; **p < 0.01, compared to the cisplatin-only group. Detailed values are as follows: For A549 cells treated with 2.5 μM cisplatin, viability was 86.90 ± 3.10 %; when treated with 60 μM Abrine plus 2.5 μM cisplatin, viability decreased to 76.42 ± 1.73 %. For A549R cells, treatment with 10 μM cisplatin resulted in a viability of 88.14 ± 3.17 %, which further decreased to 61.33 ± 4.02 % upon co-treatment with 60 μM Abrine and 10 μM cisplatin. J: Cell survival of indicated cells treated with a combination of Abrine (60 μM) and cisplatin (10 μM), and co-intervention with overexpression of SPTBN2, Fer-1, GSH, respectively, for 48 h. Cis: Cisplatin (*p < 0.05, **p < 0.01).

### 2.7 SPTBN2 inhibition enhances ferroptosis-based cisplatin therapy in vivo

**Fig. 7:**
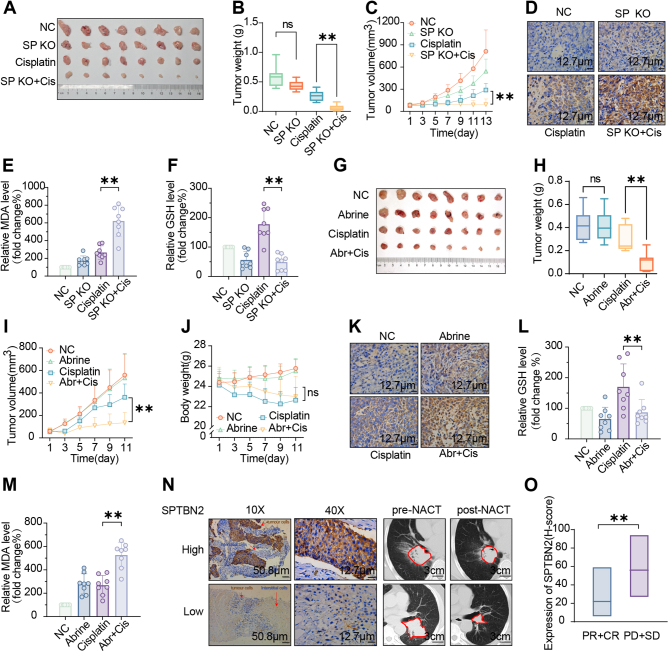
SPTBN2 inhibition enhances ferroptosis-based cisplatin therapy in vivo A-F: Subcutaneous injection of indicated A549R cells (specifically A549 R6 and SPTBN2 KO #1 sequence) into mice, followed by intraperitoneal injection of cisplatin (2.5 mg/kg, every other day) for 13 days. Tumor images (A), tumor weight (B), changes in tumor volume during treatment (C), representative immunohistochemical images of 4-HNE in tumor sections (D), MDA level in tumors (E), and GSH content in tumors (F) for each treatment group were presented. G–M: Subcutaneous injection of indicated A549R cells into mice, followed by intraperitoneal injection of drug-loaded or 5 mg/kg cisplatin every 5 days from day 7. Concurrently, Abrine (100 mg/kg) was injected intraperitoneally every other day for 11 days. Tumor images (G), tumor weight (H), changes in tumor volume during treatment (I), changes in mouse body weight during treatment (J), representative immunohistochemical images of 4-HNE in tumor sections (K), GSH content in tumors (L), and MDA level in tumors (M) for each treatment group were shown. N: Representative immunohistochemical images of SPTBN2 staining in puncture sections of patients before neoadjuvant chemotherapy and corresponding CT images of patients before and after neoadjuvant chemotherapy. O: Relationship between SPTBN2 expression level and prognosis following neoadjuvant chemotherapy (PR + CR, n = 10; PD + SD, n = 10). SP: SPTBN2; Abr: Abrine; Cis: Cisplatin. Comparisons were made using a two-tailed, unpaired Student's t-test; *p < 0.05, **p < 0.01, ***p < 0.001.

The in vivo antitumor activity of cisplatin in combination with SPTBN2 inhibition was evaluated, either by gene knockout studies or by Abrine treatment, in a xenograft mouse model of NSCLC. From day 10, mice were subjected to intraperitoneal injection of either vehicle or 2.5 mg/kg cisplatin every other day for 13 days. While SPTBN2 KO alone exhibited a modest reduction in tumor volume and weight compared to the vector control (Fig. 7A–C), this difference was not statistically significant. Notably, when combined with cisplatin, SPTBN2 KO significantly decreased tumor volume and weight compared with the cisplatin monotherapy group, without affecting body weight (Supplementary Fig. 6A). We then assessed whether SPTBN2 depletion could synergize with cisplatin to induce ferroptosis. As shown in Fig. 7. D-F, SPTBN2 KO combined with cisplatin significantly inhibited the GSH content, and increased the levels of lipid ROS products (4-HNE and MDA). Thus, the depletion of SPTBN2 enhanced ferroptosis following cisplatin treatment. Additionally, we examined the in vivo synergistic antitumor activity of Abrine and cisplatin. Starting from day 7, mice received either vehicle or 5 mg/kg cisplatin via intraperitoneal injection every 5 days. Concurrently, 100 mg/kg Abrine or vehicle was administered by intraperitoneal injection every other day for 11 days. Results demonstrated that Abrine alone had no significant effect on tumor volume and weight compared to the vehicle control. However, the combination of Abrine and cisplatin significantly reduced tumor volume and weight compared with cisplatin monotherapy, without remarkably affecting body weight (Fig. 7G–J). Abrine administration exerted no significant hepatotoxicity and nephrotoxicity, as shown by hematoxylin and eosin (HE) staining results (Supplementary Fig. 6B). The combination of Abrine and cisplatin significantly reduced GSH content (Fig. 7L) and increased those of 4-HNE (Fig. 7K) and MDA (Fig. 7M) compared with cisplatin monotherapy, indicative of the ferroptosis state.

Furthermore, immunohistochemical staining of paraffin sections of biopsy tissues obtained from 20 patients with NSCLC who underwent cisplatin-based neoadjuvant chemotherapy revealed that those with high SPTBN2 expression had a poorer response than those with low expression (Fig. 7N and O). These clinical findings align with our in vitro results, thus underscoring the pivotal role of SPTBN2 in ferroptosis and cisplatin resistance in vivo.

## 3 Discussion

Previous studies on SPTBN2 have mainly focused on neurodegenerative diseases, and its role in non-small cell lung cancer (NSCLC) is poorly understood [[32], [33], [34]]. In this study, SPTBN2 was found to function as a negative regulator of ferroptosis in NSCLC cells. Inhibition of SPTBN2 significantly attenuated cystine uptake and glutathione (GSH) synthesis, thereby heightening cell sensitivity toward ferroptosis inducers, including cisplatin, both in vitro and in vivo. This was achieved by impairing the function of System Xc^−^ on the membrane. It was reported earlier that Inhibiting SPTBN2 can disrupt the localization and impair the functions of specific membrane proteins. Armbrust et al. [35] showed that mutated SPTBN2 caused mislocalization and functional defects of mGluR1α. Similarly, Clarkson et al. [36] found that SPTBN2 was essential for recruiting and maintaining anchorin R on the dendritic membrane of Purkinje cells, with SPTBN2 mutation reducing the level of anchorin R on the membrane. Our study further elucidates the role of SPTBN2 in modulating the localization and functionality of membrane proteins involved in the ferroptosis process.

We demonstrate that SPTBN2 can bind to SLC7A11 and facilitate its interaction with Arp1, a dynactin component, for cytoplasm-to-membrane transport. This is supported by the following results: (i) SLC7A11 was localized to the membrane in normal cells but was predominantly found in the cytoplasm in SPTBN2-deficient cells; SLC7A11 overexpression did not rescue its membrane localization without SPTBN2; only co-expression of SPTBN2 and SLC7A11 restored these levels. (ii) BFA treatment indicated that SPTBN2 regulated the pre-Golgi transport of SLC7A11. (iii) Co-IP showed interaction among SPTBN2, SLC7A11, and Arp1 under normal conditions, which disappeared when SPTBN2 was absent. However, the absence of SLC7A11 or Arp1 did not affect the interaction between SPTBN2 and the other. Thus, SPTBN2 dominates in the membrane trafficking and localization of SLC7A11. Previous findings corroborate the role of SPTBN2 in facilitating protein transport. Laia Salcedo-Sicilia et al. [37] found that in SPTBN2-depleted cells, protein transport from the endoplasmic reticulum to the Golgi and post-Golgi was impaired. Holleran et al. [26]showed that the direct binding between SPTBN2 and Arp1 recruited the dynactin complex to the intracellular membrane, and provided a direct link between the microtubule motor complex and its membrane-bound cargo. Johansson et al. [38] proposed that Rab7 only activated the dynein after interacting with SPTBN2 achieved translocation from the late endosome to the negative end of the microtubule. Clarkson et al. [39] consistently showed that after SPTBN2 mutation, it could not bind to Arp1, and the transport of EAAT4 from the Golgi to the cell membrane could not be completed, resulting in accumulation in the cytoplasm. These findings align with our aforementioned hypothesis and lend support to our conclusion that SPTBN2 facilitates the transport of SLC7A11. Furthermore, we also explored whether SPTBN2 could affect the membrane localization of SLC3A2 (the auxiliary subunit of SLC7A11). The results showed that SPTBN2 neither bound to SLC3A2 nor inhibited its membrane protein expression (Supplementary Figs. 4C–E). Consequently, these results demonstrate that the interaction between SPTBN2 and SLC7A11 is the key factor in the regulation of System Xc^−^ by SPTBN2.

Network pharmacology techniques were used to identify a potential SPTBN2 inhibitor, abrine, which is a natural compound extracted from the plant *Abrus precatorius.* Abrine has various biological activities including anti-inflammatory [40], antioxidant [41], and anticancer [42]. Liang et al. [43] reported that Abrine could enhance the antitumor effect of PD-L1 by modulating the tumor immune microenvironment. Zhang et al. [44] showed that in hepatocellular carcinoma cells, Abrine induced hepatocellular carcinoma immunity and enhanced the antitumor efficacy of immune checkpoint blockade by modulating PD-L1 signaling. In this study, Abrine enhanced ferroptosis and increased the efficacy of cisplatin by inhibiting SPTBN2, both in vitro and in vivo. This finding significantly expands the scope of the biological function of Abrine to ferroptosis and NSCLC treatment. Future studies can investigate the effect of structural modification of Abrine based on its pharmacological properties, to develop more potent and less toxic inhibitors targeting SPTBN2.

**Fig. 8:**
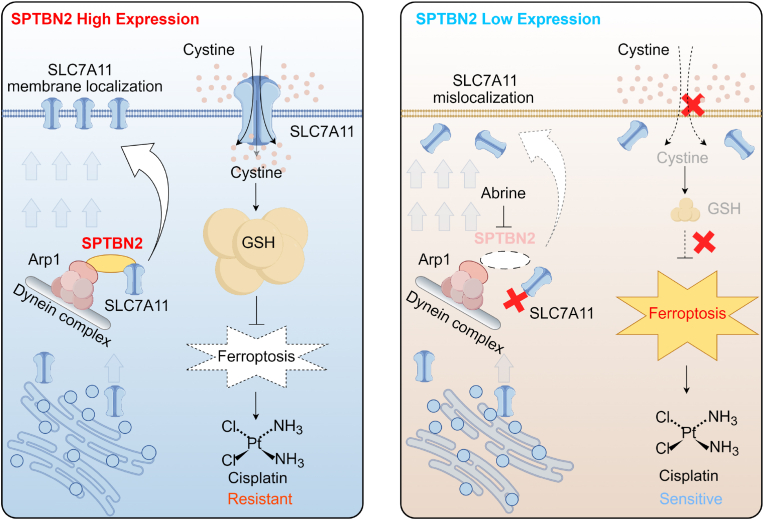
Scheme for the effect of SPTBN2 on negatively regulating ferroptosis. SPTBN2 was highly expressed in NSCLC cells, and acted as a “bridge” to connect SLC7A11 and Arp1. With the assistance of Arp1 and other dynactin complex proteins, SPTBN2 transported SLC7A11 from the cytoplasm to the cell membrane. SLC7A11 could only exert the biological function of System Xc^−^ when it was properly localized on the cell membrane, transporting cystine into the cell as a raw material for GSH synthesis, thereby inhibiting ferroptosis and making the cells resistant to cisplatin. When SPTBN2 was inhibited (such as by Abrine intervention), SLC7A11 could not effectively connect with Arp1 and the dynactin complex, and thus could not be transported to the cell membrane. The function of System Xc^−^ was inhibited, the uptake of cystine and the subsequent synthesis of GSH were reduced, and ferroptosis was induced. The increase of ferroptosis increased the sensitivity of the cells to cisplatin.

In conclusion, our study identified SPTBN2 as a suppressor of ferroptosis. Specifically, SPTBN2 preserved the function and activity of System Xc^−^ by facilitating membrane trafficking and localization of SLC7A11. Suppressing SPTBN2 expression significantly increased the sensitivity of cisplatin-resistant NSCLC cells toward cisplatin, both in vitro and in vivo, through the induction of ferroptosis (Fig. 8). Our findings provide new molecular targets and therapeutic strategies for addressing ferroptosis dysfunction and cisplatin resistance in NSCLC.

## 4 Materials and methods

### 4.1 Cell culture and regents

NSCLC cell lines (A549, H358, H460, H1299, and H838) were obtained from Cell Bank of China Science Academy in Shanghai, China. Cultivation was carried out in a 37 °C incubator with a 5 % CO2 atmosphere, using RPMI-1640 medium supplemented with 10 % fetal bovine serum and 10,000 U/mL penicillin-streptomycin. Thawed cells were passaged for a maximum of 30 days. Regular mycoplasma screenings were performed, and every 6 months, short tandem repeat profiling was conducted to confirm the authenticity of each cell line.

### 4.2 Construction of over-expression, knockdown and knockout cells

The plasmids, siRNAs and sgRNAs were transfected using jetPRIME (Polyplus transfection) according to the manufacturer's protocol. Flag-tagged SPTBN2 full-length and mutant plasmids, HA-tagged SLC7A11 plasmid were purchased from Guannan Biotechnology Co., Ltd. (Hangzhou, China), using the vector pCDH-CMV-MCS-EF1 (Puro/G418).

The siRNAs targeting SPTBN2 and Arp1 were synthesized by Genepharma Biotechnology Co., Ltd. (Shanghai, China). The sense sequences of siRNAs were as follows:

si SPTBN2#1: GCAGCCAGGAAUAUGUUCUTT
si SPTBN2#2: GGCACAAGAAGAAUCAAGATT
si Arp1#1: GGCAAUAUGUCUAUUCUAAGGTT
si Arp1#2: GGAUGAGACGCUAGAGACAGATT

The sgRNAs targeting SPTBN2 and SLC7A11 were synthesized by MailGene Biotechnology Co., Ltd. (Beijing, China). The sequences of sgRNAs were as follows:

sg SPTBN2#1: cacc GGACCTTCCTGACTCGGACT
sg SPTBN2#2: cacc GGTTGTTGATGTCACTGTAC
sg SLC7A11#1: cacc GGAGTTATGCAGCTAATTAA
sg SLC7A11#2: cacc GTGTCCACCATCTCCAAAGG

### 4.3 Regents and antibodies

Regents used in this study including Rotenone (no. S2348, Selleck), TW-37 (no. 20999, Cayman Chemical), Rapamycin (no. HY-10219, MCE),Dorsomorphin (no. 11967, Cayman Chemical), 5-Fluorouracil (no. HY-90006, MCE), Shikonin (no. HY-N0822, MCE), FIN56 (no. HY-103087, MCE), Polyphyllin VI (no. S9302, Selleck), RSL3 (no. 19288, Cayman Chemical), Z-VAD-FMK (no. HY-16658B, MCE), Necrostatin-1 (no. HY-15760, MCE), Ferrostatin-1 (no. HY-100579, MCE), Erastin (no. S7247, Selleck), iFSP1 (no. 29483, Cayman Chemical), Sorafenib (no. 10009644, Cayman Chemical), BSO (no. S9728, Selleck), Acetylcysteine (no. HY-B0215, MCE), GSH (no. HY-D0187, MCE), Cisplatin (no. HY-17394, MCE), Abrine (no. HY-N1436, MCE) were obtained from the indicated vendors.

The antibodies were as follows: SPTBN2 (Abcam, no. ab238055 for IF, IHC, WB; no.ab264178 for IP), SLC7A11 (Proteintech, 26864-1-AP for WB; Abcam, no.ab307601 for IF, IHC, WB, IP, Flow Cyt), Arp1 (Abcam, no.ab203833), Flag (Abcam, no.ab205606), HA (Abcam, no.ab9110); Tubulin (Proteintech, no.11224-1-AP), Vinculin (Proteintech, no.66305-1-Ig), Na^+^/K^+^ATPase (Beyotime, no.AF1864).

### 4.4 Xenograft tumor model

Xenograft experiments were conducted following a protocol approved by the Committee of Animal Experimental Ethical Inspection of The First Affiliated Hospital, Zhejiang University School of Medicine (Approval number: 2022-1565). This study adhered to all relevant ethical regulations concerning animal research. Female athymic nude mice (Foxn1nu/Foxn1nu), aged between four to six weeks, were employed for the experiments. Cancer cells, either 5 × 10^6^ A549 cisplatin-resistant or parental cells, were suspended in cold PBS, counted, and subcutaneously injected into each mouse. Upon reaching a tumor size of 50–100 mm^3, mice were randomly assigned to different treatment groups. Tumor volume was measured every two days, calculated using the formula: volume = length × width^2 × 0.5.

### 4.5 Patients and clinical samples

This study received approval from the Ethics Committee of Hangzhou First People's Hospital (registration number: II-T-20210907-0031-01). Tissue samples from patients diagnosed with NSCLC and corresponding para-cancerous tissues were procured at Hangzhou First People's Hospital. The retrospective collection of clinical data adhered to ethical standards. Patient clinical status was assessed according to the NCCN Clinical Practice Guidelines in Oncology for Non-Small Cell Lung Cancer, and treatment effectiveness was evaluated using the Response Evaluation Criteria in Solid Tumors 1.1.

### 4.6 Construction of cisplatin-resistant NSCLC cells

To better simulate the resistance status of cells in clinical treatment scenarios, cisplatin-resistant NSCLC cells were constructed through a gradual increase in drug concentration, starting at a low dose. Briefly, the cells were continuously co-cultured with cisplatin (IC20) at a lower dose concentration, and the cisplatin drug concentration was gradually increased after the cells adapted to the drug environment. Both resistant and parental cells were harvested at each stage for IC50 determination. This process continued until the cells demonstrated stable growth under high-concentration cisplatin treatment, resulting in the acquisition of resistant cells. The entire procedure extended over a period exceeding six months.

### 4.7 Cystine uptake assay

The Cystine Uptake Assay Kit (Dojindo Molecular Technologies, UP05) was employed for the cystine uptake assay. Cells were seeded at a density of 3000 cells per well in a black 96-well microplate and grouped according to the designated treatment protocols. Prior to the assay, the cells were cultured until they reached approximately 80 % confluence, ensuring optimal conditions for the assay. The medium was then replaced with cystine-free medium to prepare the cells for the assay. After stabilization, a cystine analog probe was introduced and incubated for 30 min. The cells were subsequently washed with cystine-free medium. The cystine uptake capacity of the cells was quantified by measuring fluorescence at λex = 490 nm and λem = 535 nm. The results were normalized to total protein using the BCA Protein Assay Kit (Thermo, 23225).

### 4.8 GSH assay

The quantification of glutathione (GSH) levels was performed using the DTNB (5,5′-Dithio-bis-(2-nitrobenzoic acid)) method (Solarbio, BC1170), with specific pre-treatment processes for tissue and cell samples. For tissue samples, tissues were homogenized on ice using a pre-chilled homogenizer at a ratio of 1:10 (tissue weight in grams to volume of reagent in mL). The homogenized samples were then centrifuged at 8000 g for 10 min at 4 °C, and the supernatant was carefully collected and stored at 4 °C until the assay was performed. For cell samples, cells were seeded at a density of 3 million cells per 100 mm culture dish and treated according to the specified experimental groups. Post-treatment, cells were harvested via trypsinization and counted. A total of 5 million cells were resuspended in 1 mL of assay buffer. To ensure complete lysis, the cell suspension underwent 2–3 freeze-thaw cycles (freezing in liquid nitrogen and thawing in a 37 °C water bath). After the final thaw, the suspension was centrifuged at 8000 g for 10 min, and the supernatant was collected and placed on ice for subsequent analysis. Following sample collection, purification with proteinase was conducted. The sample was then mixed with DTNB solution in a 96-well microplate, and the reaction was initiated by adding NADPH solution. After a 20-min incubation, the GSH level was assessed at a wavelength of 412 nm using a SpectraMax M2e spectrophotometer (Molecular Devices, San Jose, CA, USA).

### 4.9 MDA assay

The Lipid Peroxidation MDA Assay was conducted using the MDA Assay Kit (Beyotime, no.S0131 M, Beijing, China). Cells were seeded at a density of 800,000 cells per 60 mm culture dish and treated according to their designated groups. Post-treatment, cells were harvested using trypsinization and counted. A total of 3 million cells were resuspended in 0.3 mL of lysis buffer. Post-lysis, the suspension was centrifuged at 10000–12000g for 10 min to obtain a clear supernatant. In cases where centrifugation did not yield a clear supernatant, a 0.2 μm filter was used to further clarify the sample. All sample preparation steps were performed on ice or at 4 °C. Following the sample pre-treatment, the supernatants were processed as per the kit's guidelines. The samples were heated in a boiling water bath for 15 min, cooled to room temperature, and then centrifuged at 1000 g for 10 min. The absorbance of the supernatant was measured at 532 nm using an enzyme-linked immunosorbent assay. This absorbance correlates with the MDA concentration, which was calculated based on a standard curve.

### 4.10 Transmission electron microscopy

Cells were seeded at a density of 3 million cells per 100 mm culture dish and subjected to treatments as per the group protocols. Following treatment, cells were harvested via trypsinization and counted. A total of 6 million cells were resuspended in a 5 % BSA solution, followed by centrifugation. The cells were then washed with PBS, ensuring the cell pellet remained undisturbed. The supernatant was discarded, and the cell pellet was fixed overnight at 4 °C in a 2.5 % glutaraldehyde solution, with the volume exceeding the cell pellet by 20 times. The following day, cells were re-fixed in freshly prepared 2.5 % glutaraldehyde for 1 h and then in 1 % osmium tetroxide for 2 h. Cells were washed three times with PBS, each wash lasting 15 min. The cell samples underwent a stepwise dehydration process using increasing concentrations of ethanol: 50 %, 70 %, 90 %, a 90 % ethanol and acetone mixture (1:1), 90 % acetone, and finally, 100 % acetone, each step lasting 20 min. The samples were then incubated in a 2:1 and then 1:2 mixture of pure acetone and embedding resin for 3 h at room temperature and overnight, respectively. Finally, the samples were transferred to pure embedding resin and incubated for 2 h at 37 °C. The embedding process was completed with sequential incubations in an oven at 37 °C overnight, 45 °C for 12 h, and 60 °C for 24 h. Ultrathin sections of 50–60 nm thickness were prepared using an LKB-1 type ultramicrotome. Sections were double-stained with 3 % uranyl acetate and lead citrate to enhance contrast. The prepared sections were examined using a Tecnai 10 (100 kV) transmission electron microscope (FEI) located in the Electron Microscopy Core Facility at Zhejiang University. Images were captured to document the cellular structures.

### 4.11 Lipid peroxidation assay

In the lipid peroxidation assay, cells were plated at a density of 80,000 cells per well in 12-well plates and subjected to treatments as per the assigned groups. Following treatment, the cells were harvested via trypsinization and collected through centrifugation at 300*g* for 5 min. The cell pellet was washed three times with PBS, then incubated with BODIPY™ 581/591C11 dye at a final concentration of 5 μM at 37 °C with 5 % CO_2_ for 30 min. After incubation, the cells were gently washed three times with PBS to remove any unbound fluorescent dye. The cell suspension in PBS was transferred to flow cytometry tubes for analysis. The samples were evaluated using a flow cytometer, and the acquired data were further analyzed using FlowJo software. Cell subpopulations were identified based on forward scatter (FSC) and side scatter (SSC) profiles, and the proportion of the positive cell subpopulation was determined using a single-peak histogram of FL1 fluorescence signals.

### 4.12 Cell viability assay

Cell viability was assessed using the Cell Counting Kit-8 (CCK8) assay (MedChem Express, USA). Post-transfection, cells were plated at a density of 3000 cells per well in 96-well plates, and absorbance values were measured from 0 to 96 h after transfection. A solution of 100 μL RPMI 1640 medium, including 10 μL of CCK8, was added to each well, followed by a 2-h incubation. Absorbance at 450 nm was then measured using a microplate reader (Bio-Rad, USA). Each experiment was conducted in triplicate wells and repeated three times for reliability.

### 4.13 The membrane and cytoplasmic protein extraction

The membrane and cytoplasmic protein extraction kit (no. P0033) was purchased from Beyotime (Beijing, China), and the extraction was performed according to the manufacturer's instructions. In brief, cells were seeded at a density of 3 million cells per 100 mm culture dish and treated according to specified experimental groups. Post-treatment, cells were collected via trypsinization and resuspended in PBS. A minimum of 10 million cells per group were utilized for protein extraction. The cells were washed with PBS, gently resuspended in cytoplasmic lysis buffer, and incubated on ice for 10 min. This was followed by a series of freeze-thaw cycles in liquid nitrogen and a 37 °C water bath (5 cycles), and subsequent centrifugation at 700*g*, 4 °C for 10 min. The resulting supernatant, obtained after a further centrifugation at 4 °C, 14000 g for 30 min, constituted the cytoplasmic protein. The precipitate represented cell membrane fragments. This precipitate was taken, treated with Buff B (200–300 μL), vortexed for 5s every 10 min during an ice bath for 30 min, and then centrifuged at 14000 g for 5 min to obtain the membrane protein.

### 4.14 Western blotting analysis

Cells were seeded at a density of 300000 cells per well in 6-well plates and subjected to treatments as per the experimental groups. Post-treatment, total proteins were extracted using RIPA lysis buffer. A total of 20 μg of proteins were separated on 10 % SDS-PAGE and transferred to a PVDF membrane (Bio-Rad, Hercules, CA, USA). Subsequently, the membranes were blocked with 5 % non-fat milk at room temperature for 1 h, followed by an overnight incubation at 4 °C with primary antibodies. Following a wash with Tris-buffered saline containing Tween 20, the membranes underwent an additional hour of incubation with secondary antibodies at room temperature. Protein bands were visualized using the ECL system WBKLS0050 (EMD Millipore, Billerica, MA, USA) and analyzed using Bio-Rad Laboratories' Quantity One software (Bio-Rad, Hercules, CA, USA).

### 4.15 Co-immunoprecipitation

Cells were seeded at a density of 3 million cells per 100 mm culture dish and treated according to specified experimental groups. After treatment, cell lysis was performed using IP buffer, followed by centrifugation at 10,000 g for 30 min at 4 °C. The resulting cell lysates were subjected to incubation with protein G magnetic beads for 1 h at 4 °C. Immunoprecipitation reactions were initiated with equal aliquots of lysates (no-antibody, nonreactive antibody-Mouse IgG, and specific antibody). The no-antibody lysates were directly combined with loading buffer. The remaining lysates were mixed with 10 μL of protein G magnetic beads and rotated overnight at 4 °C with tightly secured caps. Subsequently, the beads underwent a thorough washing process with wash buffer (at least five times), were combined with loading buffer, and then subjected to heating at 95 °C for 5 min before proceeding to immunoblotting.

### 4.16 Histology and immunohistochemistry

In summary, Four-μm sections from paraffin-embedded tumor tissues were deparaffinized in xylene and underwent dehydration in graded alcohols. Antigen retrieval was achieved by boiling the slides in 10 mM citrate buffer in a microwave for 20 min, followed by cooling at room temperature for 30 min. Endogenous peroxidase activity was quenched by incubating the slides in 0.3 % H_2_O_2_ and subsequent washing with phosphate-buffered saline (PBS). The primary antibody was applied, and after PBS washing, the slides were incubated with the secondary antibody in a humidified chamber for 60 min, followed by 3,3-diaminobenzidine (DAB) chromogen and hematoxylin nuclear counterstaining. Positive control tissue underwent parallel staining. The staining intensity was quantified using the H-score formula: H-score = (weak intensity% × 1) + (moderate intensity% × 2) + (strong intensity% × 3).

### 4.17 Immunofluorescence

Cells were seeded at a density of 30000 cells per dish in 35 mm glass-bottom dishes (NEST, #801002) and subjected to specified condition treatments. Subsequently, cells were fixed with 4 % paraformaldehyde at room temperature for 15 min, followed by blocking with 1 % BSA at room temperature for 1 h. Incubation with the designated primary antibody occurred at 4 °C overnight. Anti-rabbit/mouse secondary antibody application transpired at room temperature for 1 h. Cell membrane staining was performed with DID for 10 min, and cell nuclei were stained with Hoechst for 15 min. Imaging was conducted using a Leica TCS SP8 confocal microscope.

### 4.18 Flow cytometry labeling of surface proteins

Flow cytometry surface protein labeling was conducted using the indirect method, involving two incubation steps: firstly with the primary antibody and subsequently with the corresponding secondary antibody. In brief, cells were seeded at a density of 300000 cells per well in 6-well plates and subjected to treatments as per the experimental groups. Post-treatment, cells were harvested, PBS-washed, and adjusted to a concentration of 1 million cells per milliliter. The cell suspension was subjected to the primary antibody, followed by a 30-min incubation in the dark at room temperature. After three PBS washes and resuspension through centrifugation, the cell suspension received a fluorescent dye-labeled secondary antibody, with another 30-min incubation in the dark at room temperature. Subsequently, the cells underwent three PBS washes, resuspension through centrifugation, and were quantitatively analyzed for surface fluorescence using flow cytometry.

### 4.19 Network-based inference method for drug-target interaction prediction

A Network-Based Inference (NBI) methodology was utilized to predict potential drug-target interactions (DTIs), as comprehensively detailed by Wu et al. [45]. Briefly, drug-related information was gathered from databases such as TCM Database, DrugBank, KEGG DRUG, IUPHAR/BPS Guide to PHARMACOLOGY, and Therapeutic Target Database to construct drug–target interaction networks. A predictive model was then developed based on the principles of NBI. The performance of the model was evaluated using 10-fold cross-validation. Subsequently, the optimal model was utilized to predict potential targets of compounds in a chemical library. Additionally, by incorporating the privileged substructure of SPTBN2, potential inhibitors of SPTBN2 were predicted.

### 4.20 Molecular docking

The X-ray crystal structures of SLC7A11(7P9V) and SPTBN2(6ANU) were retrieved from the Protein Data Bank. To ensure the accuracy of the docking results, the protein was prepared by the AutoDock Tools-1.5.7, and the water molecules were manually eliminated from the protein and the polar hydrogen was added. Docking Web Server (GRAMM) was used for protein-protein docking. The resulting protein-protein complex was also manually optimized by removing water and adding polar hydrogen by the AutoDockTools-1.5.7. Finally, the protein-protein interactions were predicted and the protein-protein interaction figure was generated by PyMOL.

### 4.21 Statistical analysis

The results were shown as the mean ± SD of at least three independent experiments. Paired *t*-test was used for comparing two paired groups, and two-sample *t*-test was applied for non-paired data. Data analyses utilized the R programming language (version 3.6.3) and GraphPad 8.0. Significance levels are indicated as *p < 0.05, **p < 0.01, and ***p < 0.001.

## Funding

This study was funded by 10.13039/501100001809National Natural Science Foundation of China (81702887), Key Laboratory of Clinical Cancer Pharmacology and Toxicology Research of Zhejiang Province (2020E10021), Zhejiang Provincial Program for the Cultivation of High-level Innovative Health Talents (ZWB-2020-18), Zhejiang Medical Key Discipline Foundation (ZWB-2018-02-03), Hangzhou Medical Key Discipline Foundation (HWF-2021-21-16), Zhejiang Provincial Natural Science Foundation (LTY21H160001, Q24H160073), Scientific and Technological Developing Scheme of Hangzhou City (20191203B49), Science Research Foundation of Zhejiang Health Bureau (2020RC026).

## CRediT authorship contribution statement

**Jun Deng:** Writing – review & editing, Writing – original draft, Visualization, Validation, Software, Project administration, Methodology, Investigation, Formal analysis, Data curation. **Xu Lin:** Validation, Resources, Project administration, Methodology, Funding acquisition. **Jiajia Qin:** Validation, Project administration, Methodology, Formal analysis, Data curation, Conceptualization. **Qi Li:** Validation, Project administration, Investigation, Formal analysis, Data curation. **Yingqiong Zhang:** Validation, Project administration, Methodology. **Qingyi Zhang:** Validation, Project administration, Methodology. **Cong Ji:** Project administration, Methodology. **Shuying Shen:** Project administration. **Yangling Li:** Methodology, Conceptualization. **Bo Zhang:** Writing – review & editing, Supervision, Methodology, Investigation, Funding acquisition, Formal analysis, Data curation, Conceptualization. **Nengming Lin:** Writing – review & editing, Supervision, Resources, Project administration, Methodology, Investigation, Funding acquisition, Formal analysis, Data curation, Conceptualization.

## Declaration of competing interest

The authors declare no conflict of interest.Author Contributions

## Figures and Tables

**figs1:**
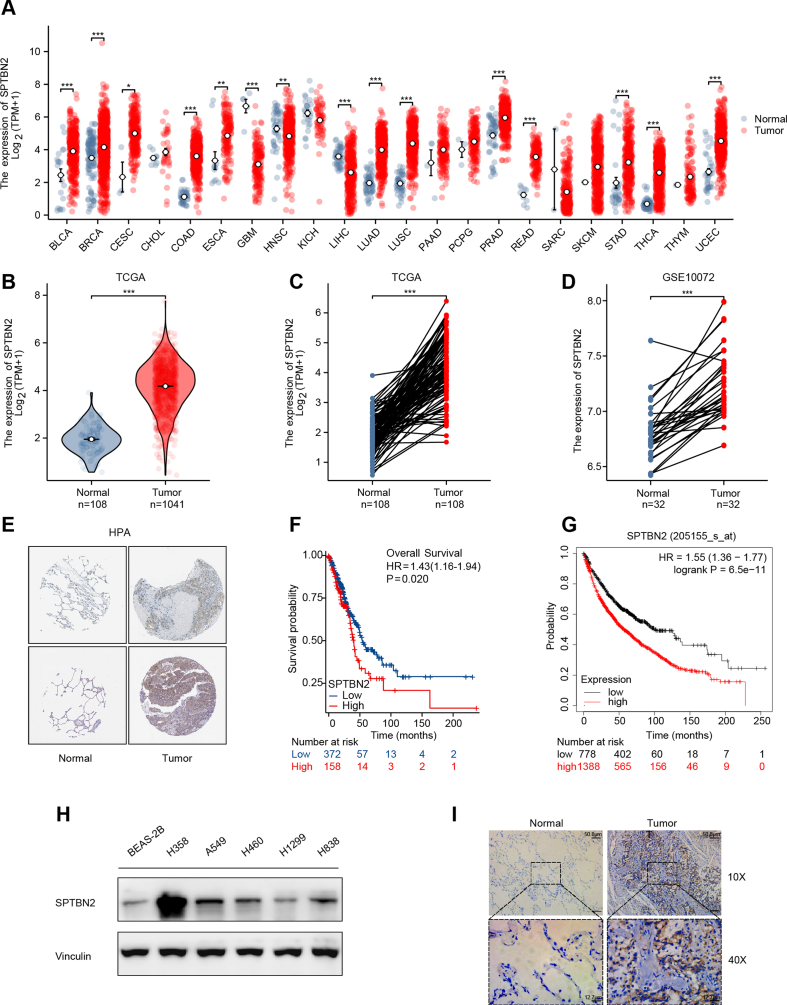


**figs2:**
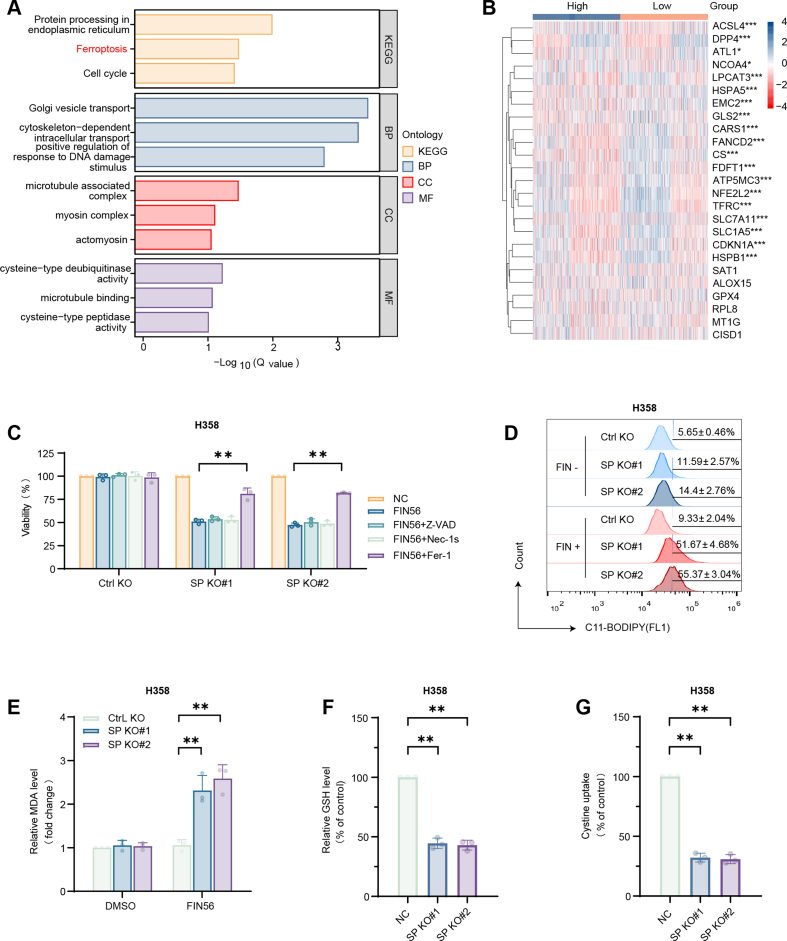


**figs3:**
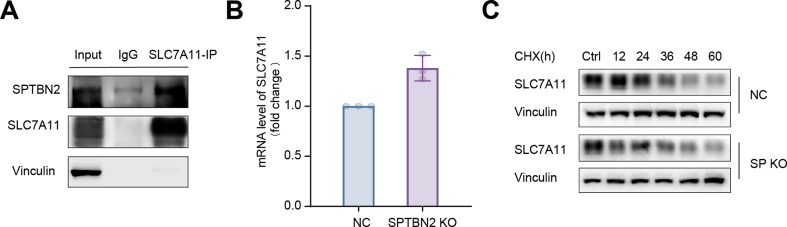


**figs4:**
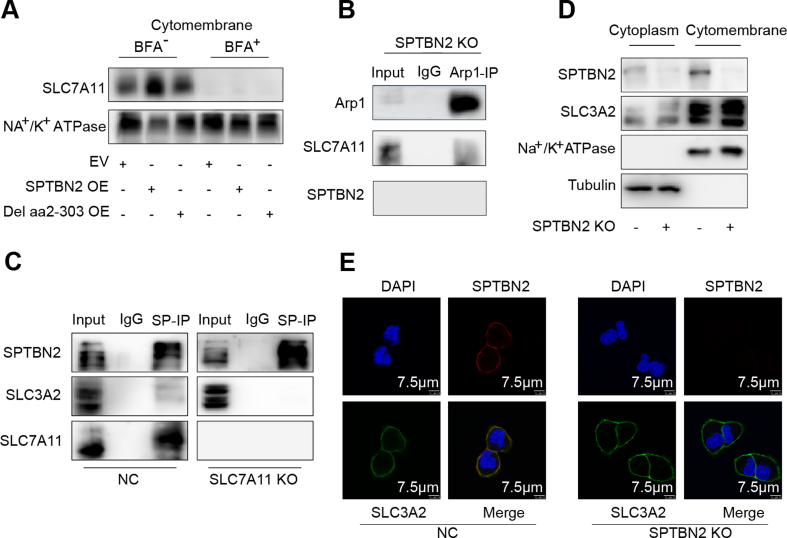


**figs5:**
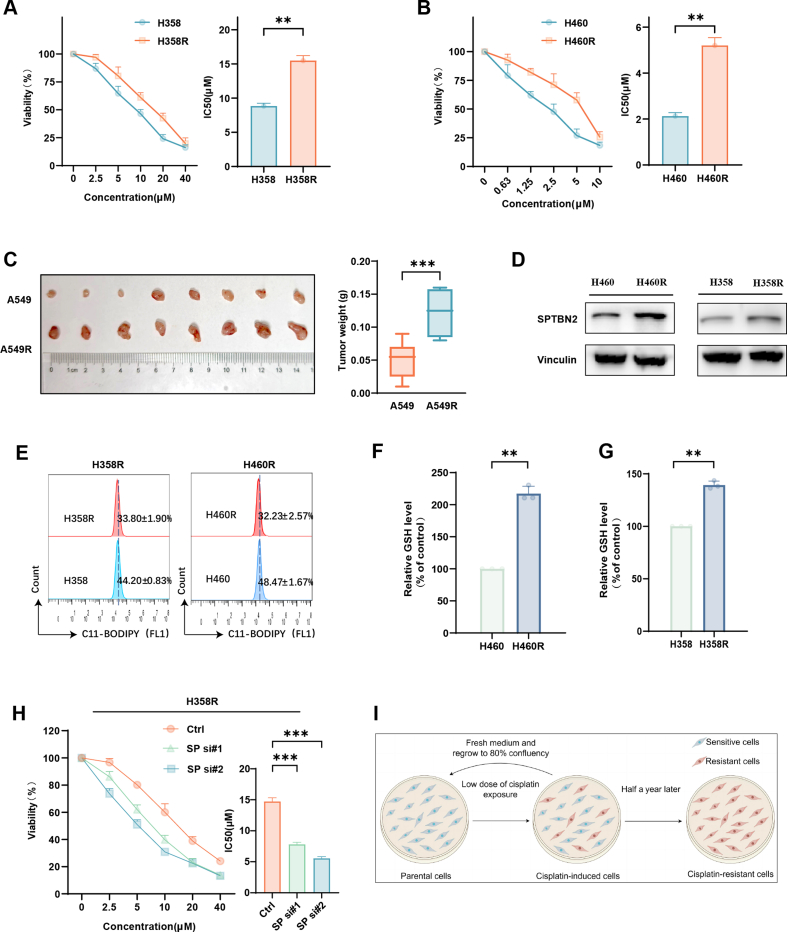


**figs6:**
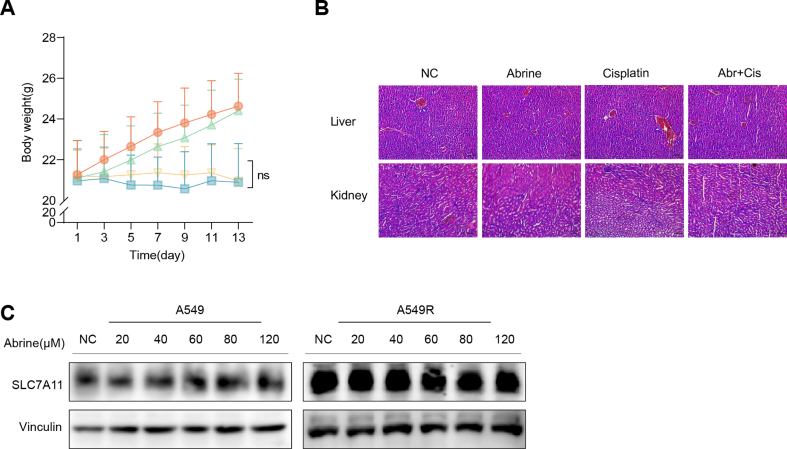


## Data Availability

Data will be made available on request.
